# CHALLENGES AND FACILITATORS TO IMPLEMENTING A PILOT HOME HEALTH PROGRAM IN THE VETERANS HEALTH ADMINISTRATION

**DOI:** 10.1093/geroni/igae098.0394

**Published:** 2024-12-31

**Authors:** Tammy Walkner, Aaron Seaman, Mary Good, Heather Benzel, Irene San Roman, Heather Davila

**Affiliations:** Iowa City VA Health Care System, Iowa City, Iowa, United States; Veterans Rural Health Resource Center, Iowa City, Iowa, United States; Iowa City VA Health Care System, Iowa City, Iowa, United States; VA Midwest Healthcare Network (VISN 23), St. Paul, Nebraska, United States; Iowa City VA Health Care System, Iowa City, Iowa, United States; Iowa City VA Health Care System, Iowa City, Iowa, United States

## Abstract

The Veterans Health Administration (VA) provides access to skilled home health (e.g. rehabilitation therapy, nursing) and home health aide services to >200,000 Veterans annually to support aging in place. Historically, these services have been provided by community-based agencies contracted with VA. In 2021, the Iowa City VA implemented the VA’s first “VA Home Health” (VAHH) program. The goals were to increase Veterans’ access to home health and improve care coordination. Given early evidence of the program’s success, VAHH expanded in 2022 to three additional sites in Iowa and South Dakota. Our team conducted semi-structured interviews with VAHH leaders (n=13) and frontline staff (interviews in progress) at the 4 program sites about their implementation experiences. Implementation barriers include: a steep learning curve; the need to define roles and create operating procedures; recruiting and hiring sufficient staff to administer and deliver the program; and challenges obtaining space and vehicles for staff. Facilitators include: staff commitment to the program; existing relationships within and outside VA; and flexibility to tailor VAHH to meet site needs. Despite similarities across sites, there were also implementation differences, primarily related to recruiting staff, hiring processes, and needs of Veterans targeted for the program (e.g. social/medical complexity). Continued evaluation is part of the implementation process, and our team will be interviewing primary care staff next to gain their perspectives. Our findings provide insights into developing and implementing a promising home health program with lessons for VA sites that may also serve as a guide for other large healthcare organizations.

